# International Journal of Molecular Science: Best Paper Award 2014

**DOI:** 10.3390/ijms15011683

**Published:** 2014-01-22

**Authors:** Stephen A. Bustin, Ian A. Nicholls, Michael Iba

**Affiliations:** 1 Postgraduate Medical Institute, Anglia Ruskin University, Chelmsford CM1 1SQ, UK; E-Mail: stephen.bustin@anglia.ac.uk; 2 Bioorganic & Biophysical Chemistry Laboratory Linnæus University Centre for Biomaterials Chemistry and School of Chemistry & Biomedicine Linnæus University, Kalmar SE-39182, Sweden; E-Mail: ian.nicholls@lnu.se; 3 Department of Pharmacology and Toxicology, Rutgers University, Piscataway, NJ 08854, USA; E-Mail: iba@eohsi.rutgers.edu

*International Journal of Molecular Science* is instituting an annual award to recognize outstanding papers in the area of chemistry, molecular physics and molecular biology published in *International Journal of Molecular Science*.

We are pleased to announce the third “*International Journal of Molecular Science* Best Paper Award” for 2014 [1,2]. Nominations were made by the Section Editors-in-Chief of *International Journal of Molecular Science* from all papers published in 2010. The awards are issued to reviews and articles separately. We are pleased to announce that the following five papers have been chosen:

## Article Award

### First Prize

**Parul Vatsa, Lisa Sanchez, Christophe Clement, Fabienne Baillieul and Stephan Dorey**

Dietary Protection Against Free Radicals: A Case for Multiple Testing to Establish Structure-activity

Relationships for Antioxidant Potential of Anthocyanic Plant Species

*Int. J. Mol. Sci.* **2010**, *11*(12), 5095–5108; doi:10.3390/ijms11125095

Available online: http://www.mdpi.com/1422-0067/11/12/5095

### Second Prize

**Junko Okuda-Shimazaki, Saiko Takaku, Koki Kanehira, Shuji Sonezaki and Akiyohshi Taniguchi**

Effects of Titanium Dioxide Nanoparticle Aggregate Size on Gene Expression

*Int. J. Mol. Sci.* **2010**, *11*(6), 2383–2392; doi:10.3390/ijms11062383

Available online: http://www.mdpi.com/1422-0067/11/6/2383

### Third Prize

**Giuseppe Calogero, Gaetano Di Marco, Silvia Cazzanti, Stefano Caramori, Roberto Argazzi, Aldo Di Carlo and Carlo Alberto Bignozzi**

Efficient Dye-Sensitized Solar Cells Using Red Turnip and Purple Wild Sicilian Prickly Pear Fruits

*Int. J. Mol. Sci.* **2010**, *11*(1), 254–267; doi:10.3390/ijms11010254

Available online: http://www.mdpi.com/1422-0067/11/1/254

## Review Award

### First Prize

**Kati Hanhineva, Riitta Törrönen, Isabel Bondia-Pons, Jenna Pekkinen, Marjukka Kolehmainen, Hannu Mykkänen and Kaisa Poutanen**

Impact of Dietary Polyphenols on Carbohydrate Metabolism

*Int. J. Mol. Sci.* **2010**, *11*(4), 1365–1402; doi:10.3390/ijms11041365

Available online: http://www.mdpi.com/1422-0067/11/4/1365

**Sonia De Pascual-Teresa, Diego A. Moreno and Cristina García-Viguera**

Flavanols and Anthocyanins in Cardiovascular Health: A Review of Current Evidence

*Int. J. Mol. Sci.* **2010**, *11*(4), 1679–1703; doi:10.3390/ijms11041679

Available online: http://www.mdpi.com/1422-0067/11/4/1679

We believe that these five exceptional papers are valuable contributions to *International Journal of Molecular Science* and the scientific research field. On behalf of the Prize Awarding Committee and the Editorial Board of *International Journal of Molecular Science*, we would like to congratulate these three teams for their excellent work. In recognition of their accomplishment, Dr. Stephan Dorey, Dr. Akiyohshi Taniguchi and Dr. Carlo Alberto Bignozzi will receive prizes of CHF 1000, CHF 800, and CHF 500, respectively, and the privilege of publishing an additional paper free of charge in Open Access format in *International Journal of Molecular Science*, after the usual peer-review procedure. Dr. Kati Hanhineva and Dr. Diego A. Moreno will receive the privilege of publishing an additional research article free of charge in Open Access format in *International Journal of Molecular Science*, after the usual peer-review procedure.

### Prize Awarding Committee

*Editor-in-Chief*, Section ‘Molecular Diagnostics’

**Prof. Dr. Stephen A. Bustin**

Postgraduate Medical Institute, Anglia Ruskin University, Chelmsford CM1 1SQ, UK

E-Mail: stephen.bustin@anglia.ac.uk

*Editor-in-Chief*, Section ‘Molecular Recognition’

**Prof. Dr. Ian A. Nicholls**

Bioorganic & Biophysical Chemistry Laboratory Linnæus University Centre for Biomaterials Chemistry and School of Chemistry & Biomedicine Linnæus University, Kalmar SE-39182, Sweden

E-Mail: ian.nicholls@lnu.se

*Editor-in-Chief*, Section ‘Molecular Toxicology’

**Dr. Michael Iba**

Department of Pharmacology and Toxicology, Rutgers University, Piscataway, NJ 08854, USA

E-Mail: iba@eohsi.rutgers.edu
